# Clinical Outcomes of Hip Abductor Repair Using Transosseous Sutures Versus Suture Anchors: A Systematic Review and Meta-analysis

**DOI:** 10.1177/23259671241290320

**Published:** 2025-01-01

**Authors:** Eduardo Portela-Parra, Elliot Sappey-Marinier, Kaitlyn Julian, Stefano A. Bini

**Affiliations:** † Department of Orthopaedic Surgery, University of California, San Francisco, San Francisco, California, USA; Investigation performed at Department of Orthopaedic Surgery, University of California, San Francisco, San Francisco, California, USA

**Keywords:** gluteus tear, gluteus medius repair, transosseous repair, suture anchor, clinical outcomes, complications, trochanteric pain syndrome, abductor tendon tears

## Abstract

**Background:**

Hip abductor tendon tears have been identified as a common cause of greater trochanteric pain syndrome. While abductor tendon tears are often managed surgically, the optimal tendon attachment technique remains controversial.

**Purpose:**

To compare the outcomes of hip abductor tendon repair between the suture anchor (SA) and transosseous suture (TS) techniques.

**Study Design:**

Systematic review; Level of evidence, 4.

**Methods:**

A literature search was performed in June 2023 in Embase, PubMed, and Web of Science databases. Studies reporting pre- and postoperative clinical outcomes of hip abductor repairs using SA or TS fixation with a minimum follow-up of 12 months were included in our analysis. From 608 studies initially identified, 21 studies (14 SA and 7 TS) with a total of 680 patients met the inclusion criteria. The PRISMA (Preferred Reporting Items for Systematic Reviews and Meta-Analyses) checklist guided the reporting and data abstraction, and the quality of the studies was assessed using the methodological index for non-randomized studies checklist. The results were presented as a narrative summary using descriptive statistics such as ranges and agreement statistics.

**Results:**

Significant pre- to postoperative improvement in pain scores and functional outcomes were reported on all included studies. The mean improvement on the Harris Hip Score/modified Harris Hip Score was 32.5 (95% CI, 28.4-36.7) for the SA technique versus 21.9 (95% CI, 6.7-37.0) for the TS technique, while mean improvement in pain according to the visual analog scale was 5.1 ± 2.3 for SA and 4.8 ± 2.2 for TS (*P* = .9). There was a trend toward statistical significance regarding retear rates, with higher rates for SA (6.7% ± 7.6%) versus TS (1.3% ± 4.7%) (*t*[13.9] = 2.0; *P* = .06).

**Conclusion:**

We observed no significant difference between SA and TS regarding improvements in patient-reported hip outcome and pain scores. However, SA trended toward a higher retear rate. Future research should propose a classification scheme that considers tear size and morphology, the extent of associated muscle degeneration, and the distance of tendon retraction to provide more context for the understanding of expected functional outcomes.

The gluteus medius and gluteus minimus muscles are hip abductor muscles and are essential for promoting pelvic stability and normal gait.^15^ Most lateral hip pain is caused by a spectrum of pathologies often grouped under the term *greater trochanteric pain syndrome*, which includes hip abductor tendinopathy or tear, trochanteric bursitis, and external snapping hip syndrome.^40^ Among these pathologies, hip abductor tendon tears are recognized as one of the most common causes of greater trochanteric pain syndrome, especially due to the thin tendinous segment of the gluteus medius tendon insertion into the greater trochanter; hip abductor tears more often affect middle-aged and elderly women without a history of hip trauma.^19^ This female predominance has been attributed to a wider female pelvis leading to a 30% larger abductor moment arm compared with the male pelvis^1,17,24,41^ as well as a smaller insertional footprint for the abductors than those in males.^44^

Management of hip abductor tendon tears that fail to improve clinically with nonoperative treatment, usually undergo surgical repair and tendon attachment.^45^ Large partial tears and full-thickness abductor tendon repairs usually require direct fixation to the greater trochanter to be effective and have traditionally been performed using either the suture anchor (SA) or the transosseous suture (TS) techniques; these procedures can be performed either open or endoscopically.^22^ However, studies on the clinical outcomes comparing these 2 surgical attachment techniques on hip abductor tendon repair are limited.

The aim of this study was to systematically review and compare studies on hip abductor tendon repair using the SA versus TS techniques. We hypothesized that hip abductor tendon repair with SA fixation would lead to superior patient-reported outcomes and fewer postoperative complications compared with TS repair.

## Methods

### Literature Search Strategy

A preliminary search was performed in June 2023 using Embase, PubMed, and Web of Science databases to select studies relating to SA and TS fixation for hip abductor tendon repair published between January 2000 and May 2023 inclusive. Two reviewers (E.P.-P. and E.S.-M.) used the phrases as Medical Subject Headings and/or text words shown in Table 1, then independently reviewed all articles identified. Each reviewer was blinded from the other reviewer's selection process. The search strategy for this study followed the PRISMA (Preferred Reporting Items for Systematic Reviews and Meta-Analyses) guidelines.^23^

**Table 1:** Search Terms by Database

Database	Query	Results
Embase	“(‘gluteal tendinopathy’/exp OR ‘gluteal tendinopathy’ OR ‘trochanteric pain syndrome’/exp OR ‘trochanteric pain syndrome’ OR ‘hip pain’/exp OR ‘hip pain’ OR ‘gluteal tendon tears’ OR ‘hip abductor tears’ OR ‘tendon reconstruction’/exp OR ‘tendon reconstruction’) AND (‘suture anchor’/exp OR ‘suture anchor’ OR ‘transosseous suture’/exp OR ‘transosseous suture’ OR ‘transosseous tunnels’ OR ‘bone tunnel’/exp OR ‘bone tunnel’)”	660
PubMed	“(gluteal tendinopathy OR trochanteric pain syndrome OR hip pain OR gluteal tendon tears OR hip abductor tears OR gluteal tendon repair OR hip abductor repair) AND (suture anchors OR transosseous tunnels OR transosseous sutures OR bone tunnels)”	58
Web of Science	“(gluteal tendinopathy OR trochanteric pain syndrome OR hip pain OR gluteal tendon tears OR hip abductor tears OR gluteal tendon repair OR hip abductor repair) AND (suture anchors OR transosseous tunnels OR transosseous sutures)”	73

### Selection Criteria

Included were case series, comparative studies, and cohort studies reporting clinical outcomes of hip abductor repair using the SA or TS attachment method with either the open or the endoscopic approach. All studies had to report ≥1 clinical outcome related to postoperative hip function with a minimum follow-up of 12 months. All included studies were written in English, involved human participants, and had full-text availability. Duplicate studies, systematic reviews, letters, conference presentations, expert opinions, cadaveric studies, studies using tendon augmentation (grafts or muscle transfers) for repairs, and editorial notes were excluded.

**Figure 1.**
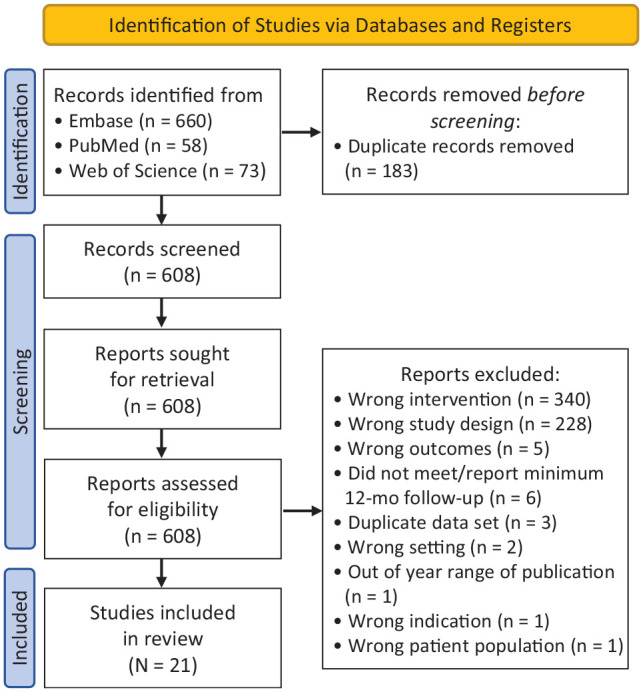
PRISMA (Preferred Reporting Items for Systematic Reviews and Meta-Analyses) flowchart of the study selection process.

Out of 608 studies initially identified, 21 studies^‡^ met the inclusion criteria for this review. Figure 1 summarizes the study selection process.

### Data Extraction

The following variables were collected: patient demographics (sample size, sex, age, body mass index), diagnostic imaging, surgical technique (approach: open vs endoscopic; attachment technique: SA vs TS), and outcome measures, including patient-reported outcome scores. Additionally, we collected the proportion of positive Trendelenburg gait, which was reported as a clinical observation. Finally, reported complication rates were collected.

Two authors (E.P.-P. and E.S.-M.) independently extracted the data, and any discrepancies were resolved through discussion and consensus between the 2 reviewers. If consensus could not be reached, a third reviewer (S.A.B.) was consulted to resolve the discrepancy.

### Quality Assessment

The quality of each study was assessed using the methodological index for non-randomized studies (MINORS).^39^ Using the items on the MINORS checklist, noncomparative studies can achieve a maximum score of 16. The categorization of noncomparative studies used in a previous systematic review^37^ was used as follows: scores 15 to 16 indicate high-quality evidence, 8 to 14 indicate fair quality, 5 to 7 indicate low quality, and 0 to 4 indicate very low-quality evidence. Two authors (E.P.-P. and K.J.) independently assigned MINORS scores to all articles collected. Discrepancies were resolved by a third arbitrator (E.S.-M.).

### Statistical Analysis

Meta-analysis of patient-reported outcomes was conducted with STATA 17 (StataCorp). Random-effects models were implemented using the restricted maximum likelihood method. The influence of improvement (mean change) in Harris Hip Score/modified Harris Hip Score (HHS/mHHS) values on the attachment technique was examined as a single moderator in the primary analysis. The heterogeneity statistics *τ*^2^, *I*^2^, and *H*^2^ were calculated for each model. According to established conventions, *I*^2^ values of 0% to 35% were considered low, 36% to 65% were considered moderate, 66% to 85% were considered substantial, and 86% to 100% were considered high levels of heterogeneity.^20^ A 2-sample weighted Student *t* test was also conducted to compare pre- with postoperative differences in pain visual analog scale (pVAS) and postoperative complication rates. Statistical significance was considered at *P* < .05.

## Results

### Study Characteristics

All 21 studies employed a case series study design; 12 studies were retrospective case series^§^, 7 studies were prospective case series,^5,9,12,30,32,42,46^ and 2 studies^29,43^ did not specify whether the data were retrospectively or prospectively collected. No comparative studies were included. Overall, 14 studies^‖^ concerned the SA technique and 7 studies^13,14,25,28,29,36,43^ reported on the TS technique. An open approach was reported in 10 studies^¶^ and an endoscopic approach in 11 studies.^#^ Of the studies using an endoscopic approach, only Meghpara et al^28^ used the TS method. The total study population was 680 patients, 336 who underwent hip abductor repair with SA and 344 patients who underwent repair with TS. The characteristics of the included studies are summarized in Table 2.

**Table 2:** Characteristics and Methodological Quality of the Included Studies (N = 21 studies, 680 patients)^*a*^

Lead Author (Year)	Study Design	Approach	Follow-up^*b*^	Sample Size, N	Female, n	Mean Age, y	Mean BMI	MINORS Score^*c*^
Suture anchor								
Voos^42^ (2009)	PCS	Endosc	19	10	8	50	NR	12
Domb^12^ (2013)	PCS	Endosc	24	15	14	58	26.2	12
McCormick^27^ (2013)	RCS	Endosc	12	10	7	66	28.8	9
Bogunovic^4^ (2015)	RCS	Endosc	24	30	27	62	NR	11
Byrd^5^ (2017)	PCS	Endosc	24	12	12	56	NR	12
Perets^32^ (2017)	PCS	Endosc	60	14	13	57	28.1	11
Hartigan^17^ (2018)	RCS	Endosc	24	25	24	54	26.8	12
Okoroha^31^ (2019)	RCS	Endosc	24	60	55	58	27.6	12
Kirby^21^ (2020)	RCS	Endosc	24	19	15	51	25.3	13
Nazal^30^ (2020)	PCS	Endosc	24	15	12	67	28.8	13
Davies^9^ (2009)	PCS	Open	12	16	15	63	NR	9
Makridis^26^ (2014)	RCS	Open	12	67	62	68	24.6	11
Barrera^2^ (2021)	RCS	Open	24	14	13	64	NR	10
Zimmerer^46^ (2021)	PCS	Open	12	29	24	60	28.00	12
Transosseous suture								
Meghpara^28^ (2021)	RCS	Endosc	24	22	21	63	29.9	10
Lübbeke^25^ (2008)	RCS	Open	12	18	13	67	28	11
Fearon^13^ (2009)	RCS	Open	12	24	24	56	NR	9
Miozzari^29^ (2010)	CS	Open	12	12	8	62	NR	11
Walsh^43^ (2011)	CS	Open	12	72	72	62	NR	10
Rajkumar^36^ (2011)	RCS	Open	12	11	6	71	NR	10
Fox^14^ (2020)	RCS	Open	60	185	152	69	NR	9

a BMI, body mass index; CS, case series; Endosc, endoscopic; MINORS, methodological index for non-randomized studies; NR, not reported; PCS, prospective case series; RCS, retrospective case series.

b Minimum follow-up in months.

c Maximum score for noncomparative studies is 16. Scores >15-16 indicate high-quality evidence, 8-14 indicate fair quality, 5-7 indicate low quality, and 0-4 indicate very low-quality evidence.

### Risk of Bias and Quality of Evidence

There were 2 studies^21,30^ with high methodological quality and 19 studies^**^ with fair methodological quality according to MINORS scores (Table 2). None of the studies collected had low or very low methodological quality. Common limitations included a lack of prospective sample size calculation, lack of blinding, and loss to follow-up of >5%.

### Preoperative Evaluation

A total of 15 studies^††^ included a description of the preceding nonoperative interventions (eg, rest, physical therapy, anti-inflammatory drugs, activity modifications, and corticosteroid injections) as a criterion for patient inclusion in the study, 4 studies^2,21,26,43^ indicated that preceding nonoperative treatment was a preoperative requirement but did not specify which interventions were applied, and 2 studies^14,25^ did not include information about preceding nonoperative management.

During preoperative evaluation, all except 1 study^36^ used imaging as a diagnostic tool for the tendon tears. However, the tears were confirmed intraoperatively. Lübbeke et al^25^ used ultrasound imaging as a diagnostic tool, and Fearon et al^13^ used ultrasound or magnetic resonance imaging.

### Outcome Scores

Among the studies collected, there were 25 different clinical outcome assessments reported (21 assessments in SA, and 14 assessments in TS), which are summarized in Table 3. The most common patient-reported outcome measures were the HHS/mHHS for hip function and pVAS (Table 4). Consequently, HHS/mHHS and pVAS were used as part of a quantitative comparison of attachment technique on studies with available preoperative and postoperative scores.

**Table 3:** Clinical Outcome Measures Used in the Included Studies by Technique and Approach^*a*^

Study (Year)	Approach	Clinical Outcome Measures
Suture anchors		
Voos^42^ (2009)	Endosc	mHHS, abductor strength, ROM
Domb^12^ (2013)	Endosc	mHHS, HOS-ADL, HOS-SS, NAHS, MRC, pVAS
McCormick^27^ (2013)	Endosc	mHHS, HOS-ADL, HOS-SS, abductor strength
Bogunovic^4^ (2015)	Endosc	mHHS, HOS-ADL, HOS-SS, pVAS
Byrd^5^ (2017)	Endosc	mHHS, iHOT-12
Perets^32^ (2017)	Endosc	mHHS, HOS-SS, NAHS, pVAS
Hartigan^17^ (2018)	Endosc	mHHS, HOS-SS, HOS-ADL, NAHS, pVAS
Okoroha^31^ (2019)	Endosc	mHHS, HOS-ADL, HOS-SS, pVAS
Kirby^21^ (2020)	Endosc	mHHS, NAHS
Nazal^30^ (2020)	Endosc	mHHS, HOS-ADL, HOS-SS, NAHS, iHOT-33, LEFS, pVAS, Trendelenburg
Davies^9^ (2009)	Open	MDP, OHS, SF-36 (PCS and MCS), pVAS, Trendelenburg
Makridis^26^ (2014)	Open	HHS, Lequesne index, significant disability, pain during stair climbing, cannot walk >1 km, pVAS
Barrera^2^ (2021)	Open	mHHS, abduction strength, Trendelenburg, pVAS
Zimmerer^46^ (2021)	Open	mHHS, UCLA, pVAS
Transosseous sutures		
Meghpara^28^ (2021)	Endosc	mHHS, NAHS, HOS-SS, SF-12, iHOT-12, VR-12, Trendelenburg, pVAS
Lübbeke^25^ (2008)	Open	HHS, pain (0-44), limp (0-11)
Fearon^13^ (2009)	Open	HHS, ODI, Trendelenburg, pVAS
Miozzari^29^ (2010)	Open	HHS, pain, limp, Trendelenburg, abductor strength
Walsh^43^ (2011)	Open	MDP
Rajkumar^36^ (2011)	Open	HHS, OHS, Trendelenburg
Fox^14^ (2020)	Open	OHS

a Endosc, endoscopy; HHS, Harris Hip Score; HOS-ADL, Hip Outcome Score–Activities of Daily Living; HOS-SS, Hip Outcome Score–Sports Subscale; iHOT-12 or -33, International Hip Outcome Tool–12 or –33; LEFS, Lower Extremity Functional Scale; MCS, Mental Component Summary; MDP, Merle d’Aubigné and Postel hip scoring system; mHHS, modified Harris Hip Score; MRC, Medical Research Council Scale for Muscle Strength; NAHS, Non-Arthritic Hip Score; ODI, Oswestry Disability Index; OHS, Oxford Hip Score; PCS, Physical Component Summary; pVAS, pain visual analog scale; ROM, range of motion; SF-12 or -36, 12-Item or 36-Item Short Form Health Survey; UCLA, University of California, Los Angeles; VR-12, Veterans RAND 12-Item Health Survey.

**Table 4:** Summary of Reported Outcomes^*a*^

Study (Year)	Measure	Outcomes^*b*^			*P*
		Preop	Postop	Δ_(Postop-Preop)_	
Suture anchors					
Voos^42^ (2009)	mHHS	NR	94 (84-100)^*c*^	NR	NR
Domb^12^ (2013)	mHHS	48.9	84.6	35.7	<.0002
	pVAS	6.8	1.4	5.4	<.001
McCormick^27^ (2013)	mHHS	NR	84.7 ± 14.5	NR	NR
Bogunovic^4^ (2015)	mHHS	55.6 ± 7.2	81.1 ± 5.6	25.5	<.0001
	pVAS	6 ± 1.8	1.7 ± 1.3	4.3	<.0001
Byrd^5^ (2017)	mHHS	42	85	43	<.001
Perets^32^ (2017)	mHHS	52.4 ± 19.9	81.2 ± 19.7	28.8	.004
	pVAS	6.2 ± 2.2	2.6 ± 2.8	3.6	.001
Hartigan^17^ (2018)	mHHS	54.9	76.2	21.3	<.001
	pVAS	7.1	2.7	4.4	<.001
Okoroha^31^ (2019)	mHHS	46.2 ± 16.5	74.6 ± 19.1	28.4	<.001
	pVAS	67.6 ± 17.6	27.3 ± 26.0	40.3	NR
Kirby^21^ (2020)	mHHS	37.7 ± 13.0	75.8 ± 18.5	38.1	<.001
Nazal^30^ (2020)	mHHS	54.3 ± 14.8	82.9 ± 16.4	28.6	<.001
	Trendelenburg	15 (100)	0 (0)	15 (100)	NR
	pVAS	5.4 ± 1.8	2.4 ± 3.0	2.93	.02
Davies^9^ (2009)	Trendelenburg	11 (68.8)	5 (31.2)	6 (54.5)	NR
	pVAS	7	2	5	.002
Makridis^26^ (2014)	HHS	50.4 ± 8.0	87.9 ± 15.5	37.5	<.001
	pVAS	8.7 ± 1.1	1.7 ± 2.7	7	<.001
Barrera^2^ (2021)	mHHS	59.1 ± 7.1	92.7 ± 4.6	33.6 ± 6.5	.001
	Trendelenburg	10 (71)	0 (0)	10 (100)	<.001
	pVAS	7.4 ± 1.0	1.3 ± 1.3	6.1 ± 0.9	<.001
Zimmerer^46^ (2021)	mHHS	28.6 ± 13.7	71.6 ± 28.3	43	<.0001
	pVAS	8.9 ± 1.1	3.14 ± 2.6	5.76	<.0001
Transosseous sutures					
Meghpara^28^ (2021)	mHHS	55.9	75.4	19.5	.005
	pVAS	5.9	3.6	2.3	.009
Lübbeke^25^ (2008)	HHS	NR	74.4 ± 15.9	NR	NR
Fearon^13^ (2009)	HHS	NR	74 (50-92)^*d*^	NR	NR
	pVAS	85 (75-100)^*d*^	9 (0-25)^*d*^	71 (50-92)^*d*^	.0002
Miozzari^29^ (2010)	HHS	38.8 ± 7.4	75.1 ± 11.9	36.3	.02
	Trendelenburg	12 (100)	2 (16.7)	10 (83.3)	NR
Rajkumar^36^ (2011)	HHS	77.4 (55-87)^*c*^	86.97 (79-96)^*c*^	9.57	NR
	Trendelenburg	11 (100)	3 (27.3)	7 (63.6)	NR

a HHS, Harris Hip Score; mHHS, modified Harris Hip Score; NR, not reported; Postop, postoperative; Preop, preoperative; pVAS, pain visual analog scale.

b Outcomes are reported as mean or mean ± SD unless otherwise indicated. Trendelenburg outcomes are reported as No. (%) of patients with positive sign.

c Reported as mean (range).

d Reported as median (interquartile range).

### Meta-analysis

#### Change in HHS/mHHS Score

**Figure 2.**
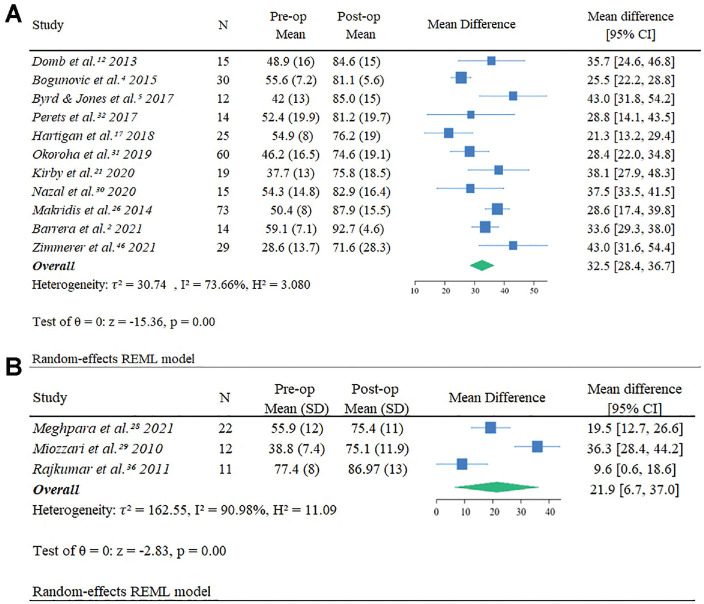
Forest plots showing the difference in mean pre- and postoperative Harris Hip Score/modified Harris Hip Score scores between studies using (A) suture anchor and (B) transosseous suture fixation. Postop, postoperative; Preop, preoperative; REML, restricted maximum likelihood.

Overall, 11 studies on SA^2,4,5,12,17,21,26,30-32,46^ (n = 306 patients) reported pre- and postoperative HHS/mHHS scores, with an overall improvement of 32.5 (95% CI, 28.4-36). The heterogeneity was substantial (τ^2^ = 30.74; *I*^2^ = 73.66%; *H*^2^ = 3.080) (Figure 2A). There were 3 studies on TS^28,29,36^ (n = 87 patients) that reported pre- and postoperative HHS/mHHS scores, with an overall improvement of 21.9 (95% CI, 6.7-37.0). The heterogeneity was high (τ^2^ = 162.55; *I*^2^ = 90.98%; *H*^2^ = 11.09) (Figure 2B). Despite the SA studies’ showing a larger mean improvement in HHS/mHHS scores compared with the TS studies, the substantial overlap in confidence intervals indicated no statistically significant difference between improvement scores.

#### Change in pVAS Score

Overall, 10 SA^2,4,9,12,17,26,30,31,32,46^ and 2 TS^13,28^ studies including 331 patients (285 from SA, and 46 from TS) reported the pre- to postoperative change in pVAS scores. There was no statistically significant difference in mean improvement in pVAS scores between SA (5.1 ± 2.3) and TS (4.8 ± 2.2) (*t*[1.1] = 0.14; *P* = .91).

#### Complication and Retear Rates

A total of 13 SA^‡‡^ and 5 TS^14,28,29,36,43^ studies involving 578 (276 SA and 302 TS) patients described the presence or absence or postoperative complications, which included retear, wound infection, deep vein thrombosis, pulmonary embolism, hip joint subluxation, and hematoma (Table 5). There was no statistically significant difference in postoperative complication rates between SA (8.3% ± 9.4%) and TS studies (9.0% ± 9.5%) (*t*[8.4] = −0.15; *P* = .88). In a subanalysis comparing the overall complication rate between endoscopic versus open approaches, there was a trend toward statistical significance (endoscopic: 4.1% ± 6.4%; open: 10.6% ± 9.3%; *t*[12.8] = −1.78; *P* = .09).

**Table 5:** Summary of Postoperative Complications^*a*^

Study (Year)	Approach	Complication Rate, %	Complications	
			General	Hip Specific
Suture anchors				
Voos^42^ (2009)	Endosc	0	None	None
Domb^12^ (2013)	Endosc	7	None	1 superficial infection
McCormick^27^ (2013)	Endosc	0	None	None
Bogunovic^4^ (2015)	Endosc	7	None	2 “repair failures”
Byrd^5^ (2017)	Endosc	0	None	None
Perets^32^ (2017)	Endosc	0	None	None
Hartigan^17^ (2018)	Endosc	0	None	None
Okoroha^31^ (2019)	Endosc	NR	NR	NR
Kirby^21^ (2020)	Endosc	0	None	None
Nazal^30^ (2020)	Endosc	0	None	None
Davies^9^ (2009)	Open	31	None	4 retears, 1 deep wound infection
Makridis^26^ (2014)	Open	16	None	11 “surgical failures”
Barrera^2^ (2021)	Open	0	None	None
Zimmerer^46^ (2021)	Open	14.0	None	3 retears, 1 wound infection
Transosseous sutures				
Meghpara^28^ (2021)	Endosc	18	None	Spasms and lateral thigh numbness, 3 retears
Lübbeke^25^ (2008)	Open	NR	NR	NR
Fearon^13^ (2009)	Open	NR	NR	NR
Miozzari^29^ (2010)	Open	17	Acetabular loosening	1 hip joint subluxation
Walsh^43^ (2011)	Open	19	6 DVT, 1 PE	1 pressure sore, 1 wound hematoma, 1 retear, 1 fracture GT, 1 wound infection
Rajkumar^36^ (2011)	Open	0	None	None
Fox^14^ (2020)	Open	4	DVT	None

a DVT, deep vein thrombosis; Endosc, endoscopic; GT, greater trochanter; NR, not reported; PE, pulmonary embolism.

There was a trend toward statistical significance in retear rates between SA (6.7% ± 7.6%) and TS (1.3% ± 4.7%) (*t*[13.9] = 2.0; *P* = .06). In a subsequent subanalysis, there was no significant difference in retear rates between endoscopic (2.9% ± 5.1%) and open approaches (4.3% ± 7.7%) (*t*[12.3] = −0.47; *P* = .65).

### Rehabilitation Protocol

Overall, 11 studies^§§^ (8 SA and 3 TS^25,28,36^) implemented abduction braces for range restriction. There were 14 studies^‖ ‖^ (11 SA and 3 TS^28,29,36^) that used a 6- to 8-week partial weightbearing postoperative protocol, 2 SA studies^27,30^ used immediate full weightbearing, and 4 TS studies^13,14,25,43^ used a 3- to 6-week nonweightbearing protocol. Byrd and Jones^5^ did not specify whether the protocol was partial or nonweightbearing, but they implemented walking aid for 8 weeks.

## Discussion

The major finding of this review was that there were no significant differences in HHS/mHHS improvement, pVAS improvement, and complication rates between the SA versus TS attachment methods for hip abductor repair. In effect, studies on both SA and TS techniques reported substantial improvement in functional hip scores and pain, as well as correction of Trendelenburg gait following repair of abductor tendon tears.

Despite finding no statistically significant differences in the complication rates between the SA and TS techniques, this difference trended toward statistical significance in a subanalysis comparing endoscopic versus open approaches. In addition, postoperative deep vein thrombosis was only found in studies using the open approach. In a prior systematic review evaluating endoscopic versus open approaches, Chandrasekaran et al^6^ concluded that endoscopic repair was associated with fewer wound complications, with similar improvements in functional outcomes and pain reduction compared with open repair. Therefore, any potential differences in the rate of postoperative complications might be secondary to the surgical approach used or patient selection, not the tendon attachment technique.

For decisions on which attachment technique should be implemented, previous studies have suggested that the morphology of the tear should indicate the type of fixation technique selected. Davies et al^10^ reported the use of SA for grade 1 to 2 tears and TS for grade 4 to 5 tears. Kenanidis et al^20^ proposed an algorithm to guide the use of TS versus SA fixation based on the Goutallier Fuchs classification,^16^ in which tears with a classification >2 should undergo augmented repair (such as tendon graft, dermal allograft, or muscle transfer) using the TS technique.^19^ For hip abductor tears with poor tendon quality (high degree of fatty infiltration and retraction), tendon augmentation and muscle transfer techniques using the vastus lateralis, tensor fascia latae, or gluteus maximus have been reported with both TS and SA techniques.^20^ Synthetic grafts and allografts have been successfully implemented in the repair of functioning tendons with limited fatty infiltration.^45^

The comparative biomechanical strength of these 2 surgical attachment techniques is poorly studied, as is their relative clinical success. The hip joint experiences high loads, around 4 to 5 times body weight during normal walking and up to 8 times body weight when stumbling.^3,8^ Biomechanical cadaveric studies on the hip model have shown SAs to have pullout/tensile strengths ranging between 200 and 400 N.^11,18,35^ No biomechanical studies were found showing tensile strengths of TS on the hip. Moreover, there are no comparative studies on tensile strength or clinical outcomes of hip abductor repair using TS versus SA. In contrast, there are several studies comparing these 2 attachment techniques in other muscle groups/joints, such as those in the rotator cuff/shoulder^7,34^ and the quadriceps tendon/knee.^33,38^ These studies showed mixed results in terms of the biomechanical superiority of TS versus SA techniques.

### Strengths and Limitations

There are several strengths to this study. This is the only review comparing studies using SA versus TS, offering a meta-analysis of HHS/mHHS and reporting improvement in pVAS scores, as well as total postoperative complication and retear rates.

There are also several important limitations to this study. As noted previously, there is a considerable amount of heterogeneity in terms of patient selection, postoperative care protocols, the reported data, the clinical outcome scores selected, and how perioperative data were collected and reported. This heterogeneity makes comparison of clinical outcomes between the 2 primary attachment techniques or the surgical approaches difficult. This finding might partially explain the lack of previous meta-analyses comparing clinical outcomes after hip abductor repair, unlike studies focusing on rotator cuff, patellar tendon, and quadricep tendon repairs.^33,34,38^ Another limitation of this study is the inclusion criterion of a minimum 12-month follow-up, as it could be too early to identify the true retear rate. In addition, it is unclear if a comparison of clinical outcomes reported at 12 months and a 60-month follow-up is appropriate. Finally, the sample size for most studies was relatively small compared with those seen in studies reporting results for tendon tears elsewhere in the body, suggesting that more data need to be published in this area.

## Conclusion

All studies in the current review reported significant substantial improvement in pain scores and functional outcomes. We observed no statistically significant difference between SA and TS attachment regarding improvements in hip assessment and pain scores. However, we observed a trend toward a significant difference between the techniques in retear rates, with SA fixation having a higher retear rate. There was substantial variability in the outcome assessment tools used, which highlights the need for more consistent reporting of clinical outcomes after abductor tendon repair. Future research should propose a classification scheme that considers tear size and morphology, the extent of associated muscle degeneration, and the distance of tendon retraction to provide more context for the understanding of expected functional outcomes.

## Notes

‡ References 2, 4, 5, 9, 12, 13, 14, 17, 21, 25-32, 36, 42, 43, 46.

§ References 2, 4, 13, 14, 17, 21, 25-28, 31, 36.

‖ References 2, 4, 5, 9, 12, 17, 21, 26, 27, 30-32, 42, 46.

¶ References 2, 9, 13, 14, 25, 26, 29, 36, 43, 46.

# References 4, 5, 12, 17, 21, 27, 28, 30-32, 42.

** References 2, 4, 5, 9, 12-14, 17, 25, 26-29, 31, 32, 36, 42, 43, 46.

†† References 4, 5, 9, 12, 13, 17, 27, 28, 29, 30-32, 36, 42, 46.

‡‡ References 2-5, 9, 12, 17, 21, 26, 27, 30, 32, 42, 46.

§§ References 2, 4, 9, 12, 17, 21, 26, 28, 29, 31, 32, 36, 42, 46.

‖‖ References 2, 4, 9, 12, 17, 21, 26, 28, 29, 31, 32, 36, 42, 46.
